# Supplementary material for: Left–right-alternating theta sweeps in entorhinal–hippocampal maps of space
Source: Nature. 2025 Feb 3;639(8056):995–1005. doi: 10.1038/s41586-024-08527-1 (PMC11946909; doi:10.1038/s41586-024-08527-1)
Supplement: Supplementary file 1 — Reporting Summary [file 41586_2024_8527_MOESM1_ESM.pdf]

nature portfolio

Corresponding author(s): Abraham Z. Vollan
Edvard I. Moser

Last updated by author(s): Nov 23, 2024

Reporting Summary

Nature Portfolio wishes to improve the reproducibility of the work that we publish. This form provides structure for consistency and transparency in reporting. For further information on Nature Portfolio policies, see our Editorial Policies and the Editorial Policy Checklist.

Statistics

For all statistical analyses, confirm that the following items are present in the figure legend, table legend, main text, or Methods section.

n/a	Confirmed	
☐	☒	The exact sample size (*n*) for each experimental group/condition, given as a discrete number and unit of measurement
☐	☒	A statement on whether measurements were taken from distinct samples or whether the same sample was measured repeatedly
☐	☒	The statistical test(s) used AND whether they are one- or two-sided<br>*Only common tests should be described solely by name; describe more complex techniques in the Methods section.*
☐	☒	A description of all covariates tested
☐	☒	A description of any assumptions or corrections, such as tests of normality and adjustment for multiple comparisons
☐	☒	A full description of the statistical parameters including central tendency (e.g. means) or other basic estimates (e.g. regression coefficient) AND variation (e.g. standard deviation) or associated estimates of uncertainty (e.g. confidence intervals)
☐	☒	For null hypothesis testing, the test statistic (e.g. *F*, *t*, *r*) with confidence intervals, effect sizes, degrees of freedom and *P* value noted<br>*Give P values as exact values whenever suitable.*
☒	☐	For Bayesian analysis, information on the choice of priors and Markov chain Monte Carlo settings
☒	☐	For hierarchical and complex designs, identification of the appropriate level for tests and full reporting of outcomes
☐	☒	Estimates of effect sizes (e.g. Cohen's *d*, Pearson's *r*), indicating how they were calculated

Our web collection on statistics for biologists contains articles on many of the points above.

Software and code

Policy information about availability of computer code

Data collection	Commercial software: Motive (OptiTrack) version 2.2.0; MATLAB (MathWorks) version r2019b<br>Open-source software: SpikeGLX (https://billkarsh.github.io/SpikeGLX) versions 20190724 and 20190919

Data analysis	Commercial software: MATLAB (MathWorks) version r2020b, Python version 3.7<br><br>Open-source code (for MATLAB): Kilosort version 2.5 (https://github.com/MouseLand/Kilosort); UMAP version 1.4.1 (https://www.mathworks.com/matlabcentral/fileexchange/71902-uniform-manifold-approximation-and-projection-umap); CircStat toolbox (https://github.com/circstat/circstat-matlab); Chronux toolbox (http://chronux.org/); Latent manifold tuning model (https://github.com/waq1129/LMT); CCH deconvolution (https://github.com/EranStarkLab/CCH-deconvolution); Code for reproducing the analyses in this article are available at Zenodo, DOI: placeholder (will-be-provided-at-proofs-stage).<br><br>Open-source Python packages:<br>numpy 1.18.1<br>scanpy 1.7.2<br>pandas 1.1.5<br>anndata 0.7.8<br>scipy 1.4.1<br>scikit-learn 0.22.2<br>matplotlib 3.3.4<br>DeepLabCut 2.3.10

For manuscripts utilizing custom algorithms or software that are central to the research but not yet described in published literature, software must be made available to editors and reviewers. We strongly encourage code deposition in a community repository (e.g. GitHub). See the Nature Portfolio guidelines for submitting code & software for further information.

Data

Policy information about availability of data

All manuscripts must include a data availability statement. This statement should provide the following information, where applicable:

- Accession codes, unique identifiers, or web links for publicly available datasets
- A description of any restrictions on data availability
- For clinical datasets or third party data, please ensure that the statement adheres to our policy

The datasets generated during the current study are available at EBRAINS, DOI: https://doi.org/10.25493/R5FR-EDG.

Research involving human participants, their data, or biological material

Policy information about studies with human participants or human data. See also policy information about sex, gender (identity/presentation), and sexual orientation and race, ethnicity and racism.

Reporting on sex and gender	N/A
Reporting on race, ethnicity, or other socially relevant groupings	N/A
Population characteristics	N/A
Recruitment	N/A
Ethics oversight	N/A

Note that full information on the approval of the study protocol must also be provided in the manuscript.

Field-specific reporting

Please select the one below that is the best fit for your research. If you are not sure, read the appropriate sections before making your selection.

☒ Life sciences ☐ Behavioural & social sciences ☐ Ecological, evolutionary & environmental sciences

For a reference copy of the document with all sections, see nature.com/documents/nr-reporting-summary-flat.pdf

Life sciences study design

All studies must disclose on these points even when the disclosure is negative.

Sample size	Samples included all available cells that matched the classification criteria for the relevant cell type.
Data exclusions	Cells with low firing-rates (<0.1Hz or 0.025Hz) were excluded from analyses because of their unsuitability for spike-train analysis.
Replication	In the results text we indicate for each result the number of animals or grid modules in which the effect was found. Multiple recordings were done in each animal and the recording session with the best unit yield and behavioral performance was included in the study.

Randomization	The study did not involve any experimental subject groups; therefore, random allocation did not apply and was not performed.
Blinding	The study did not involve any experimental subject groups; therefore, experimenter blinding did not apply and was not performed.

Reporting for specific materials, systems and methods

We require information from authors about some types of materials, experimental systems and methods used in many studies. Here, indicate whether each material, system or method listed is relevant to your study. If you are not sure if a list item applies to your research, read the appropriate section before selecting a response.

Materials & experimental systems

n/a	Involved in the study
☒	☐ Antibodies
☒	☐ Eukaryotic cell lines
☒	☐ Palaeontology and archaeology
☐	☒ Animals and other organisms
☒	☐ Clinical data
☒	☐ Dual use research of concern
☒	☐ Plants

Methods

n/a	Involved in the study
☒	☐ ChIP-seq
☒	☐ Flow cytometry
☒	☐ MRI-based neuroimaging

Animals and other research organisms

Policy information about studies involving animals; ARRIVE guidelines recommended for reporting animal research, and Sex and Gender in Research

Laboratory animals	Long Evans rats, male and female, age 3-4 months (300-500 g)
Wild animals	None
Reporting on sex	Findings apply to both sexes (17 males, 1 female rat). Sex-based analyses were not performed.
Field-collected samples	None
Ethics oversight	Protocols approved by the Norwegian Food Safety Authority (FOTS ID 18011)

Note that full information on the approval of the study protocol must also be provided in the manuscript.

Plants

Seed stocks	N/A
Novel plant genotypes	N/A
Authentication	N/A
